# Canopy Plants Safeguard the Taxonomic and Phylogenetic Diversity of Recruiting Plants Worldwide

**DOI:** 10.1111/ele.70447

**Published:** 2026-08-25

**Authors:** G. Gleiser, J. M. Alcántara, J. Bascompte, J. L. Garrido, A. Montesinos‐Navarro, G. B. Paterno, A. Valiente‐Banuet, M. Verdú

**Affiliations:** ^1^ Centro de Investigaciones Sobre Desertificación (CIDE‐CSIC) Carretera de Moncada‐Náquera Km 4.5 Moncada Valencia Spain; ^2^ Instituto de Investigaciones en Biodiversidad y Medioambiente (INIBIOMA) Universidad Nacional del Comahue‐CONICET San Carlos de Bariloche Río Negro Argentina; ^3^ Departamento de Biología Animal, Biología Vegetal y Ecología Universidad de Jaén Jaén Spain; ^4^ Department of Evolutionary Biology and Environmental Studies University of Zurich Zurich Switzerland; ^5^ Departamento de Microbiología del Suelo y Sistemas Simbióticos Estación Experimental del Zaidín (EEZ‐CSIC) Granada Spain; ^6^ Biodiversity, Macroecology and Biogeography, Faculty of Forest Sciences and Forest Ecology University of Göttingen Göttingen Germany; ^7^ Departamento de Ecología de la Biodiversidad, Instituto de Ecología Universidad Nacional Autónoma de México México DF México; ^8^ Centro de Ciencias de la Complejidad Universidad Nacional Autónoma de México, Ciudad Universitaria México DF México

**Keywords:** aridity gradient, competition, facilitation, Hill diversity, phylogenetic diversity, plant community ecology, plant recruitment, plant–plant interactions

## Abstract

Plant recruitment—the transition from seed dispersal to seedling establishment—is a vulnerable stage strongly shaped by canopy plants, which modify microclimates, soils, and biotic interactions relative to open ground. Using a global database covering 142 communities across ten terrestrial biomes, we examined canopy effects on taxonomic and phylogenetic diversity at global and local scales, quantified with Hill numbers (*q* = 0, 1, 2) to weight rare, common, and dominant species. Globally, canopy microsites hosted five times more exclusive species and over three times more evolutionary history than open ground. Locally, canopies buffered aridity's negative effects, sustaining higher diversity. For *q* = 1, open ground had higher diversity under low aridity, but under dry conditions canopies promoted rare, stress‐sensitive species alongside dominants. These patterns show that canopy plants act as microrefugia, preserving both species richness and evolutionary history, highlighting their critical role as guardians of plant diversity under increasing climatic aridity.

## Graphical abstract

We provide a global synthesis on how canopy plants shape both taxonomic and phylogenetic diversity during recruitment, the most climatically sensitive stage of the plant life cycle. Drawing from explicit plant–recruit interactions across 142 communities spanning ten biomes, our analysis reveals that canopy plants act as universal microrefugia. By sheltering rare, common, and dominant species, canopy plants preserve disproportionate evolutionary history relative to open ground, behaving as critical global custodians of plant diversity.

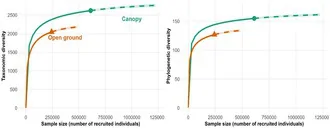

## 1 Introduction

The diversity of plant communities varies profoundly in space, and the study of such variation is a foundational pursuit in plant ecology, as diversity is tightly linked to ecosystem functioning (Wagg et al. 2017; Hautier et al. 2018). Climate is a major driver of plant diversity (Hawkins et al. 2003; Kreft and Jetz 2007), with aridity standing as a primary abiotic pressure that promotes shifts in species composition, functional traits, and community structure (Harrison et al. 2020; Gleiser et al. 2025). Previous research has largely focused on patterns of adult plant diversity along gradients of aridity, temperature, and soil properties (e.g., Berdugo et al. 2020). However, we still lack a comprehensive understanding of how aridity shapes plant community diversity during the crucial early stages of establishment, when seedling recruitment and survival determine long‐term community composition (Shackelford et al. 2021). This gap is particularly important given the strong contrasts in microclimatic conditions across microhabitats ‐such as open ground versus canopy‐covered areas which can substantially influence recruitment dynamics and early community assembly (Soliveres et al. 2014). While adult plant diversity offers a contemporary snapshot of a plant community, quantifying the diversity of the early plant life stages is crucial for assessing its regeneration potential. This is especially important given the pervasive threats that plant communities face, such as habitat fragmentation, land‐use change, fires, droughts, biological invasions, and the broader impacts of climate change (Franklin et al. 2016; Pausas and Keeley 2021).

Plant recruitment, the successful transition from seed dispersal to seedling establishment, is a demographic bottleneck for plant communities (Harper 1977; Valdez et al. 2019), and is notably more sensitive to the projected effects of climate change, such as increasing aridification, than the adult stage (Walck et al. 2011). The success of plant recruitment largely depends on the availability of suitable microhabitats for seedling establishment, which are determined by the ecological requirements of each species (Gómez‐Aparicio 2008). These requirements define the recruitment niche (Grubb 1977), which, in many cases, is provided by canopy species–adult plants which modify microclimatic conditions, soil properties and biotic interactions relative to the surrounding bare ground. The buffering effect provided by canopies against extreme climatic conditions is particularly evident in drylands, where recruitment is often limited by high temperatures, intense solar radiation, and scarce water availability. In such environments, canopy plants enable the establishment of species that otherwise would be excluded from those communities (Valiente‐Banuet and Verdú 2013; O'Brien et al. 2019; Vega‐Álvarez et al. 2019). Conversely, canopy plants may hinder recruitment by creating unsuitable microhabitat conditions (May and Ash 1990; Weedon and Facelli 2008), or by depressing—and in some cases entirely preventing—the recruitment of certain species underneath them (Alcántara et al. 2018). Consequently, the diversity of recruiting species within a community is expected to be shaped not only by broad climatic factors but also, at finer spatial scales, by the outcomes of plant–plant interactions, which are themselves context‐dependent (Maestre et al. 2009; Alcántara et al. 2025), species‐specific (Callaway 1998; Castillo et al. 2010; Paterno et al. 2016) and might change with ontogeny (Miriti 2006; Paterno et al. 2016).

Beyond ameliorating understory microenvironments, canopy plants–particularly those from harsh environments–offer more heterogenous recruitment niches by increasing variation in light, temperature or soil fertility compared to bare ground (Moro et al. 1997; Soliveres et al. 2011; Sánchez‐Martín et al. 2024). This heterogeneity in the regeneration niches is expected to allow the recruitment of species with different traits (Paterno et al. 2016; Fagundes et al. 2022), likely stemming from different lineages. Additionally, competitive exclusion among recruits tends to occur between closely related lineages; therefore, surviving recruits are more likely to belong to phylogenetically distant lineages (Castillo et al. 2010). Together, these processes enhance the phylogenetic diversity—that is, the evolutionary history—of the species pool recruiting beneath canopy plants. As regeneration niches are often evolutionarily conserved (Valiente‐Banuet and Verdú 2007), the phylogenetic diversity of a pool of recruits is a good proxy of its functional diversity (i.e., functional trait diversity; Tucker et al. 2018). Therefore, the joint estimation of the taxonomic and phylogenetic diversity of the recruits provides an integrative measure of community diversity, as these two complementary dimensions may diverge in their responses to environmental and biotic filters (Devictor et al. 2010; Swenson 2011; Hähn et al. 2024).

A global synthesis of over 10,000 experimental and observational cases of plant–plant interactions indicates that competition is more prevalent than facilitation across plant communities, although this pattern varies considerably across biomes and ontogenetic stages (Yang et al. 2022). Despite the dual role of canopy plants in promoting both facilitation and competition, both processes can contribute to increases in taxonomic and phylogenetic diversity (facilitation by expanding the range of suitable microhabitats and competition by limiting dominance and promoting niche partitioning). This is supported by co‐occurrence analyses of adult plants, which highlight the role of canopy species as safeguards of these diversity components—with stronger effects on taxonomic diversity in alpine ecosystems and on phylogenetic diversity in drylands (Bashirzadeh et al. 2022). However, community‐level studies that go beyond pairwise experimental designs are needed to provide the broader ecological context in which plant–plant interactions occur. In particular, focusing on the recruitment stage—the most limiting phase in the plant life cycle—is essential to understand the mechanism underlying the generation and maintenance of diversity in plant communities, as co‐occurrence analyses based on adult individuals conflate different ontogenetic stages and may obscure key mechanisms. Moreover, integrating data across multiple biomes is crucial to understand how these interactions shape biodiversity under contrasting environmental conditions.

In this study, we address these gaps by estimating the taxonomic and phylogenetic diversity of recruiting plant species from a wide array of plant communities spanning different biomes and aridity levels worldwide. Our main objective is to evaluate whether canopy plants enhance different facets of diversity—specifically taxonomic and phylogenetic—compared to open ground, and to assess whether this potential effect varies along an aridity gradient from humid to arid communities. We hypothesize that canopy plants will increase both taxonomic and phylogenetic diversity of recruiting species relative to open ground, and that this positive effect will be stronger in more arid communities, where facilitative interactions are expected to be more pronounced. We used abundance‐based diversity measures through Hill numbers, which allow differential weighting of rare versus common species (Chao et al. 2014). This approach is particularly relevant given that facilitation has been suggested to disproportionately benefit rare species (Soliveres et al. 2015), and thus, using Hill numbers enables a more nuanced assessment of how aridity and microhabitat conditions affect not only species richness but also community evenness and dominance patterns among recruiting species. By providing a more comprehensive and ecologically meaningful understanding of the diversity of the pool of recruiting species, this approach offers valuable insights into the regeneration potential and thus resilience of the plant communities, a topic that is gaining increasing relevance given the accelerating aridification scenario projected for many regions, especially drylands (Mirzabaev et al. 2019).

## 2 Methods

### 2.1 Database and Phylogenetic Hypotheses

**FIGURE 1:**
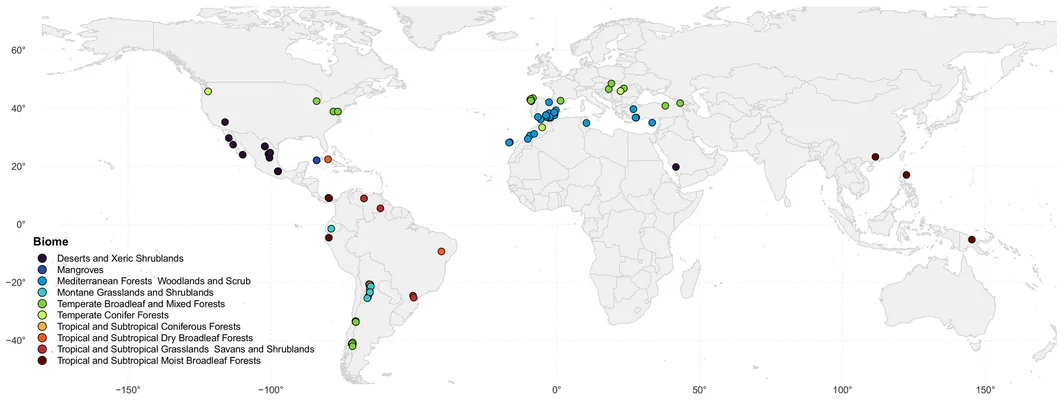
Geographic distribution of study sites. Each point represents one of the 142 plant communities, colour‐coded by biome.

We obtained data from the RecruitNet database, which contains records on plants recruiting in open ground or in association with canopy plants (recruiting microsites hereafter). This database gathers data from studies spanning 143 plant communities from both tropical and temperate regions from five continents, covering 10 of the 14 types of terrestrial biomes (Verdú et al. 2023; Figure 1). In these studies, field sampling was conducted by applying one of the following protocols: (1) the Recruitment Network (RN) protocol, in which sampling units (e.g., plots or transects) are defined, and where all plants (canopies and recruits) are identified; (2) the paired Canopy‐Open (pCO) protocol, in which a focal canopy plant is identified, and all plants recruiting in association with this focal canopy and in a nearby open ground of the same area are recorded; and (3) the Georeferenced Plot (GP) protocol, where canopy‐recruit interactions were inferred from the locations of georeferenced plants. We excluded one study site because it had not registered plants recruiting in the open ground. This yielded a total of 142 plant communities, with total areas ranging from 14 to 500,000 m^2^ (23,796 ± 81,558 m^2^, mean and SD Table S1). This huge variation in sampling effort was associated with the type of studied community (Verdú et al. 2023). Despite this variation, coverage—a metric reflecting how comprehensively a community has been sampled (Chao and Jost 2012)—was high, with link coverage averaging 0.95, ranging between 0.60 and 1.00, with most studies (86.71%) reaching a coverage above 0.90 (Verdú et al. 2023). To represent the evolutionary relationships among all the recruiting species in the RecruitNet database, we used the megaphylogeny derived in Gleiser et al. (2025). Briefly, the phylogeny was generated using the R package V.PhyloMaker2 (Jin and Qian 2022), which builds large‐scale trees from species lists based on the GBOTB.extended.TPL.tre megaphylogeny (Smith and Brown 2018). Prior to analysis, species names were standardized to match those in the reference tree by correcting spelling errors and verifying accepted names according to World Flora Online (WFO 2023). For genera absent from the megaphylogeny, we incorporated phylogenetic affinities obtained from published sources to improve the placement of missing taxa. The final tree was produced using the options “S3” (for inserting absent species) and “nodes.info.1.TPL” (for handling non‐monophyletic genera). This comprehensive phylogeny was then pruned for each plant community according to the list of recruiting species recorded. From this phylogeny, we also derived pairwise phylogenetic distances between species, representing the evolutionary time separating them (in millions of years, Myr). These distances were used as a basis for quantifying phylogenetic diversity within the studied communities.

### 2.2 Estimation of Taxonomic and Phylogenetic Diversity

We estimated the taxonomic and phylogenetic diversity of the pool of species recruiting in open ground and under canopies using Hill numbers (Hill 1973). This approach for measuring diversity considers not only species richness but also the evenness of the communities in terms of species abundance, and has preferred mathematical properties in comparison with previous approaches (i.e., it conforms to the replication principle; Chao et al. 2014). More specifically, Hill numbers incorporate the most widely used diversity indexes inside a unified expression that includes a parameter “q” that determines the sensitivity of the estimation to abundance unevenness. Although q can take any value, three q values are frequently considered because of their association with the three most popular diversity indexes. For *q* = 0, diversity equals species richness as relative abundances are not taken into account. For *q* = 1, species are weighted by their frequencies and diversity approaches the exponential of Shannon entropy (measuring the number of common species), and for *q* = 2, the contribution of rare species to diversity is negligible and diversity equals the inverse Simpson concentration index (measuring the number of dominant species, that is, of those species with greater abundances). More broadly, as q increases, Hill numbers decline most significantly in communities with greater unevenness among species. Diversity estimated through Hill numbers then measures the effective number of equally abundant species (or entities under a broader framework, see below), allowing straightforward comparisons, which are not possible using traditional diversity indexes.

Beyond taxonomic diversity, Hill numbers have been extended to consider other dimensions of biodiversity (Chao et al. 2014), such as phylogenetic diversity, which takes into account the amount of shared evolutionary history among species. Instead of considering the species as the unit of interest, phylogenetic Hill numbers are measured based on phylogenetically equally distinct entities, with a phylogenetic entity being a unit‐length branch segment. Species frequencies are incorporated by weighting each entity by the corresponding branch abundance, obtained by summing the relative abundance of all species descended from that branch. Phylogenetic Hill numbers are associated with Faith's phylogenetic diversity (a phylogenetic generalisation of species richness; Faith 1992) when *q* = 0 (i.e., the abundances are not considered), while high values of q give more weight to those nodes in the phylogeny with higher relative abundances. The specific cases where *q* = 1 and *q* = 2 are related to Allen's et al. phylogenetic entropy (a phylogenetic generalisation of Shannon entropy; Allen et al. 2009) and to Rao's quadratic entropy (a phylogenetic generalisation of the Simpson's index; Rao 1982) respectively (Chao et al. 2010). Taxonomic and phylogenetic diversity based on Hill numbers are directly comparable, as both are expressed in units of effective numbers of equally abundant species or evolutionary lineages (Chao et al. 2014). For instance, the theoretical maximum of phylogenetic diversity in a community corresponds to its taxonomic diversity, which would occur if all species were maximally divergent—that is, shared no evolutionary history. In such a case, each species would contribute equally as one effective number of species and one unit of branch length.

At the global (γ) scale, we assessed taxonomic and phylogenetic diversity by pooling all communities across the globe. Specifically, we quantified the total taxonomic and phylogenetic diversity (TD and PD) of all species recruiting under canopies and in open ground across all communities, considering *q* = 0. This analysis reflects the number of species and lineages of the global species pool associated with each recruiting microsite, irrespective of species' local abundances or their distribution among communities. To determine whether the observed phylogenetic diversity associated with each microsite was greater or lower than expected given its species richness, we calculated the standardized effect size of phylogenetic diversity (SES.PD) using the R package picante (Kembel et al. 2010) and a null model that shuffled taxa labels across the tips of the phylogeny (taxa. labels) with 9999 randomizations. *p* values indicate the quantile position of the observed PD within the null distribution, with *p* < 0.05 or *p* > 0.95 representing significantly lower or higher PD than expected by chance. In addition, we repeated all analyses using only species exclusive to each microsite, allowing us to disentangle patterns driven by true microsite specialisation from those influenced by generalist species that recruit in both canopy and open‐ground environments, and thus providing a more robust assessment of microsite‐specific taxonomic and phylogenetic diversity.

At the local (α) scale, we evaluated within‐community patterns of diversity by estimating the taxonomic and phylogenetic Hill diversities (*q* = 0, 1 and 2) of species recruiting under canopies and in open ground separately for each community.

All the diversity estimations were made by fitting sample size–based rarefaction and extrapolation curves using the iNEXT3D function in the R package iNEXT.3D (Chao et al. 2021). This approach accounts for differences in sampling area among communities. The asymptotic estimation represents the expected diversity when sample coverage approaches 1, meaning a completely sampled community.

To assess whether phylogenetic uncertainty in the phylogenies used to estimate phylogenetic diversities could produce any biases, we built two more phylogenies using the scenarios “S1” and “S2” proposed in Jin and Qian 2019 and recalculated the asymptotic phylogenetic diversity values. The strength of the correlation between the diversity estimates derived from different input phylogenies was used to assess how sensitive our analyses are to different phylogenetic hypotheses.

### 2.3 Diversity in Relation to Recruiting Microsite and Aridity

We investigated how the diversity of recruiting plants differs between canopy and open ground microsites across contrasting levels of aridity. To quantify the level of aridity, we used the aridity index (AI), which is defined as the ratio of precipitation (P) to potential evapotranspiration (PET), the latter being a measure of the transfer of water from both soil and plant canopy to the atmosphere. Larger AI values thus indicate lower aridity levels. This index provides a quantitative measure of water availability and, thus, constitutes a continuous measure of climatic stress for plants. Discrete aridity categories have nonetheless been derived from the AI, with different dryland categories defined for those regions in which AI < 0.65 (UNEP 1997). To estimate the aridity index for each community, we downloaded a raster file with a 30 arc‐seconds resolution from Chelsa V2.1 (Karger et al. 2021) and used the R package “raster” (Hijmans 2023) to extract the corresponding values.

### 2.4 Statistical Analyses

We ran general linear mixed models to assess how Hill diversities change according to the recruiting microsite (canopy vs. open ground) and aridity level, with aridity being defined as 1‐aridity index to simplify interpretation (i.e., increasing values indicating higher aridity levels). These models were constructed separately for taxonomic and phylogenetic Hill diversities of order *q* = 0, 1 and 2. Aridity, recruiting microsite, and their interaction were treated as fixed effects, and the plant community as a random effect. We ran separate models that also included the number of canopy species in each community to account for potential confounding effects of aridity with the heterogeneity in recruitment niches provided by different canopy plants. Diversities were log‐transformed to achieve normality of residuals. We ran both linear and quadratic models, to account for a possible non‐linear relationship between diversity and aridity.

To test whether taxonomic and phylogenetic diversity exhibited different responses to aridity, we compared the slopes of TD and PD along the aridity gradient. Three separate linear models were fitted, one for each Hill number (*q* = 0, 1, 2), using a global model that included both diversity types and their interaction with aridity; the interaction term tested for differences in slopes between TD and PD. All models were run in R version 4.3.3 (R Core Team 2024) using the R packages lme4 (Bates et al. 2015) and lmerTest (Kuznetsova et al. 2017).

## 3 Results

**FIGURE 2:**
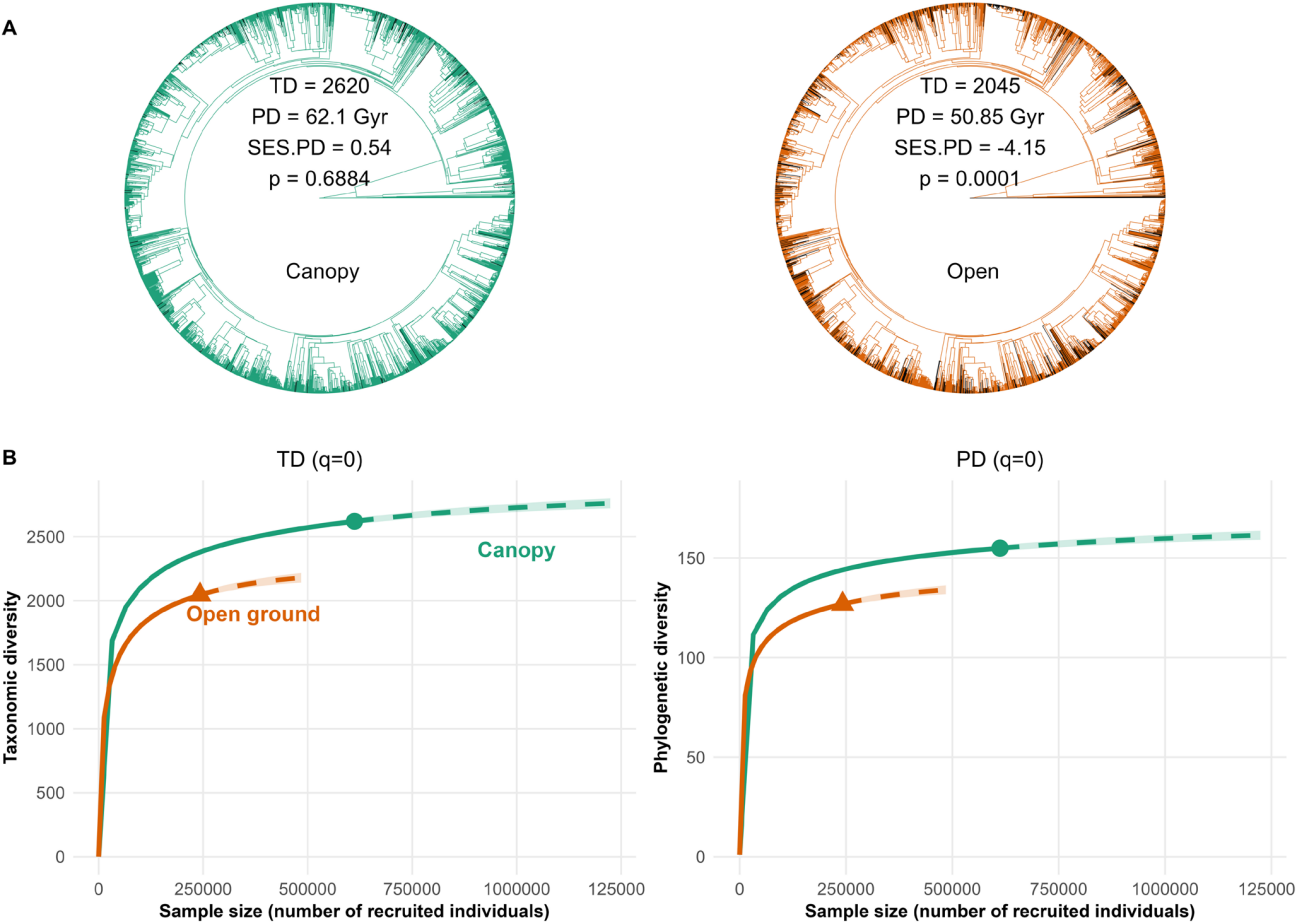
Taxonomic (TD) and phylogenetic (PD) diversity of recruiting species across microsites at the global scale. (A) Phylogenetic trees of species recruiting under canopy (left, green) and open ground (right, orange). Coloured branches indicate species present in the microsite; black branches are absent from that microsite but present in the global pool. Species occurring in both microsites appear in both trees. TD and PD values are shown within each tree, along with the standardized effect size of PD (SES.PD) and *p* values indicating whether observed PD is higher or lower than expected given species richness. While PD under canopy plants does not differ from random expectations, PD in open ground is lower than expected. (B) Sample‐size–based rarefaction and extrapolation curves comparing taxonomic diversity (left) and phylogenetic diversity (right) for species recruiting under canopies (green) and in open ground (orange) at *q* = 0. Solid lines represent observed and rarefied diversity, dashed lines indicate extrapolated values, and shaded areas show 95% confidence intervals (*N* = 30 randomizations). Patterns are qualitatively unchanged when analyses are restricted to species recruiting exclusively in one microsite, thereby excluding generalist species that recruit in both environments (Figure S1).

At the global (γ) scale, we recorded 2746 recruiting species, and both their taxonomic and phylogenetic diversity were higher under canopy microsites than in open ground (Figure 2). Across all communities, taxonomic diversity was ~28% higher under canopies, with 2620 species recruiting under canopies and 2045 in open ground. These totals include many species that occur in both microsites. When separating unique and shared species, 701 species (26%) recruited exclusively under canopies, 126 species (5%) recruited exclusively in open ground, and 1919 species (69%) recruited in both microsites. This strong dominance of canopy‐exclusive and shared species highlights the disproportionate contribution of canopies to overall recruitment diversity (Figure 2A; Figure S1A). Species recruiting under canopies also accounted for a markedly greater amount of evolutionary history—62.1 billion years, compared with 50.9 billion years for open ground recruits. The standardized effect size of phylogenetic diversity did not differ from random expectations under canopies (SES.PD = 0.54, *p* = 0.6884), whereas open‐ground microsites showed markedly lower phylogenetic diversity than expected by chance (SES.PD = −4.15, *p* = 0.0001; Figure 2A). A similar trend appeared for exclusive species: SES.PD for species recruiting exclusively under canopies did not differ from random expectations (SES.PD = 0.04, *p* = 0.4964; Figure S1A), while exclusive species in open ground exhibited marginally lower‐than‐expected phylogenetic diversity (SES.PD = −1.54, *p* = 0.0608; Figure S1A). Thus, at the global scale, canopies supported five times more exclusive species and over three times more phylogenetic diversity than open ground, revealing their disproportionate role in preserving plant taxonomic diversity and evolutionary history. These patterns remained after accounting for differences in sampling effort (Figure 2B; Figure S1B).

At the local (α) scale, defined as diversity within individual communities rather than pooled across them, species richness followed the same pattern as at the global scale, with more species recruiting under canopies than in open ground (Table 1a). Back‐transforming the asymptotic taxonomic diversities (Table 1a), 25.6 ± 2.0 species recruited under canopies, compared to 18.7 ± 1.4 species in open ground (Hill order *q* = 0), representing roughly 37% higher richness beneath canopies. The effective number of species accounting for relative abundances (*q* = 1 and *q* = 2) was also higher under canopies than in open ground: 9.9 ± 0.6 versus 7.6 ± 0.5 for *q* = 1, and 6.7 ± 0.4 versus 5.8 ± 0.5 for *q* = 2. Canopy microsites also supported higher phylogenetic diversity than open ground across all Hill orders (Table 1b). For *q* = 0, approximately 9.9 ± 0.6 lineages recruited under canopies compared to 8.7 ± 0.4 in open ground (back‐transformed means ± SE in Table 1b). When species abundances were taken into account, the effective number of lineages declined in both microsites (3.7 ± 0.2 vs. 3.4 ± 0.2 and 2.6 ± 0.1 vs. 2.4 ± 0.1 lineages recruiting under canopy vs. open ground for *q* = 1 and *q* = 2, respectively; back transformed means ± SE in Table 1b), yet remained significantly higher under canopies, indicating greater lineage diversity even at higher Hill orders. Species recruiting in both microsites were the most numerous, averaging 27 ± 3 species and contributing 1832 ± 132 million years of phylogenetic diversity. Canopy‐only recruits were fewer (6.5 ± 1.1 species) but still maintained substantial evolutionary history (1020 ± 128 million years), whereas open‐only recruits were rare (0.97 ± 0.27 species) and contributed less phylogenetic diversity (430 ± 105 million years). These results underscore how canopy‐associated species disproportionately contribute to species richness and evolutionary history, with local patterns summing to shape global diversity.

**TABLE 1:** Mean log‐transformed asymptotic diversities and their standard errors at different Hill orders of species recruiting in open ground versus canopy‐covered spaces. Diversity differences between microsites were compared with paired *t*‐tests.

(a)				
Hill order	Taxonomic diversity			*p*
	Open ground	Under canopies	*t* (df)	
*q* = 0	2.93 ± 0.08	3.22 ± 0.08	4.11 (141)	< 0.0001
*q* = 1	2.03 ± 0.07	2.29 ± 0.06	5.22 (141)	< 0.0001
*q* = 2	1.75 ± 0.08	1.90 ± 0.06	2.43 (141)	< 0.05

(b)				
	Phylogenetic diversity			*p*
	Open ground	Under canopies	*t* (df)	
*q* = 0	2.17 ± 0.05	2.30 ± 0.06	2.99 (135)	< 0.005
*q* = 1	1.23 ± 0.05	1.32 ± 0.05	4.14 (135)	< 0.0005
*q* = 2	0.89 ± 0.04	0.95 ± 0.04	3.57 (135)	< 0.001

Taxonomic and phylogenetic diversities were positively correlated in both recruiting microsites, but the strength of the relationship decreased with increasing Hill order (Table S2). In open ground, correlations ranged from *r* = 0.874 for *q* = 0 to *r* = 0.474 for *q* = 2, while under canopies they ranged from *r* = 0.859 to *r* = 0.352 (all *p* < 0.001). This decline reflects that abundant species tend to be more closely related, reducing phylogenetic diversity when species abundances are considered. Indeed, across all communities, the five most abundant species exhibited, on average, shorter phylogenetic distances among them (235 ± 5.7 Myr) than the five least abundant species (258 ± 6.5 Myr). This difference was statistically significant (paired *t*‐test: *t* = −3.35, df = 142, *p* = 0.001), indicating that dominant recruit species belong to a more restricted set of related lineages.

**FIGURE 3:**
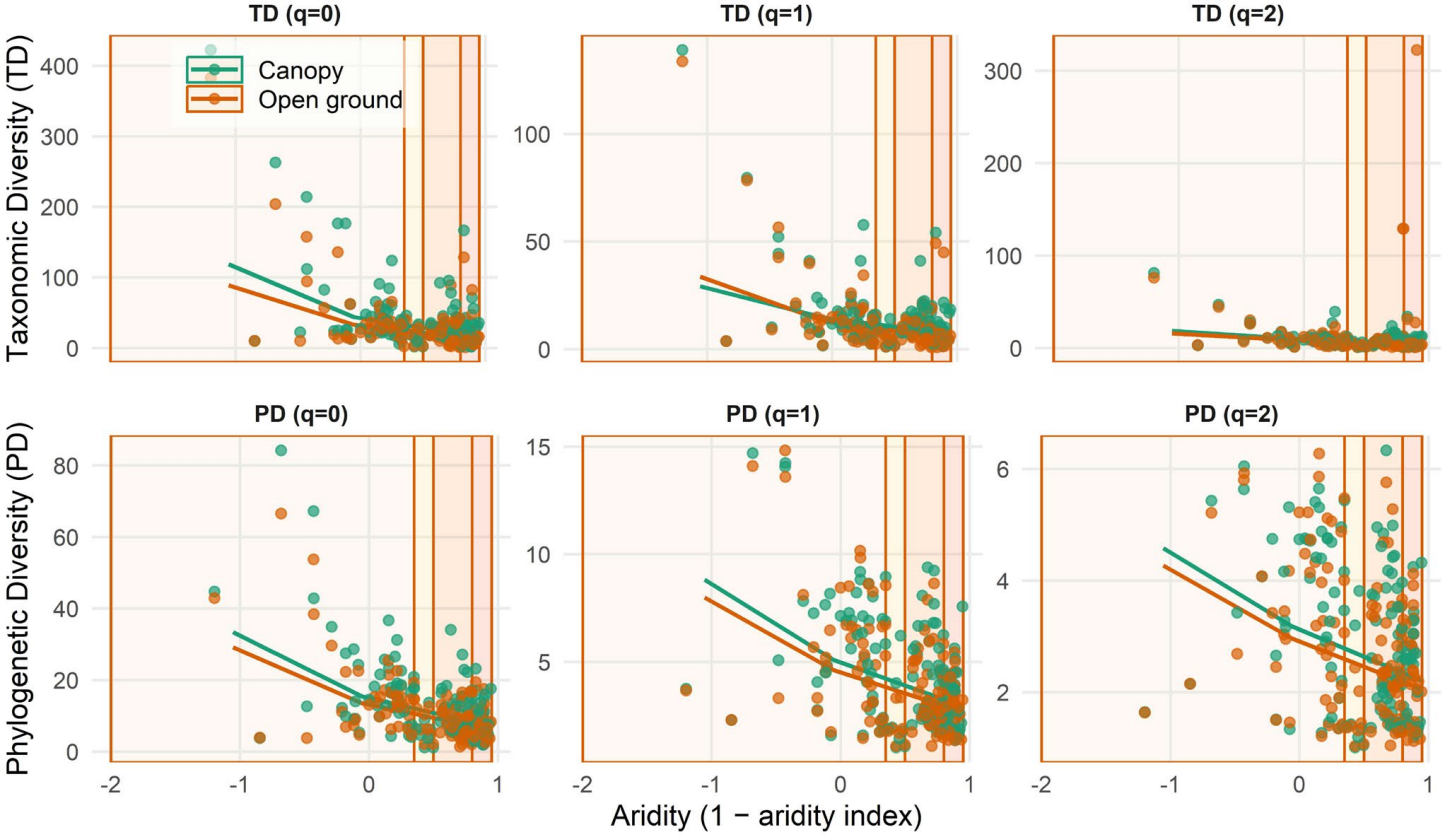
The taxonomic (top) and phylogenetic (bottom) diversities of recruiting plants decrease with aridity. The taxonomic diversities shown are the number of species (Hill order *q* = 0), the number of common species (*q* = 1) and the number of dominant species (*q* = 2). The phylogenetic diversities shown are the number of phylogenetic entities (*q* = 0), the number of common phylogenetic entities (*q* = 1), and the number of dominant phylogenetic entities (*q* = 2; see Methods). Coloured background bands from left to right indicate aridity classes: Humid, dry subhumid, semiarid and arid. Lines represent model predictions for each microsite type (canopy vs. open ground).

While canopies generally supported higher taxonomic and phylogenetic diversity than open‐ground microsites, this difference was influenced by aridity for taxonomic diversity of order *q* = 1, where a significant interaction between microsite and aridity was observed. In contrast, for *q* = 0 and *q* = 2, canopies maintained consistently higher diversity regardless of aridity (Figure 3; Table 2a). This indicates a generally positive effect of canopy plants on total species numbers and the coexistence of dominant taxa, largely independent of aridity. For *q* = 1, however, the microsite–aridity interaction showed that open ground supported higher diversity under low aridity, but this pattern reversed in drier environments, suggesting that the advantage of canopy plants in promoting an even species distribution becomes stronger under harsh, arid conditions. The results did not change after including the number of canopy species in each community as a potential confounding factor with aridity (Table S4).

**TABLE 2:** Linear mixed models testing the effects of aridity (considered here as 1‐aridity index) and microsite (canopy vs. open ground) on the diversity of the plant recruits. Both the taxonomic (a) and phylogenetic (b) Hill diversities were analysed. Plant communities were incorporated in the models as a random effect. The interaction term was removed from models where it was not statistically significant.

(a)									
	Taxonomic diversity								
	*q* = 0			*q* = 1			*q* = 2		
	Est ± SE	df	*t*	Est ± SE	df	*t*	Est ± SE	df	*t*
Intercept	3.7 ± 0.1	175.4	35.7**	2.6 ± 0.1	194	27.1**	2.2 ± 0.1	172.3	23.9**
Microsite (Open ground)	−0.3 ± 0.1	141	−4.1**	−0.1 ± 0.1	140	−1.6	−0.1 ± 0.1	141	−2.4*
Aridity	−1.1 ± 0.2	140	−6.5**	−0.7 ± 0.2	194	−4.6**	−0.6 ± 0.2	140	−4.6**
Microsite × Aridity				−0.3 ± 0.1	140	−2.1*			
Marginal *R* ^2^	0.20			0.20			0.11		
Conditional *R* ^2^	0.60			0.73			0.59		

(b)									
	Phylogenetic diversity								
	*q* = 0			*q* = 1			*q* = 2		
	Est ± SE	df	*t*	Est ± SE	df	*t*	Est ± SE	df	*t*
Intercept	2.7 ± 0.1	156.7	33.4**	1.6 ± 0.07	143.3	22.3**	1.1 ± 0.1	143.5	26.5**
Microsite (Open ground)	−0.1 ± 0.1	135.5	−3.0**	−0.09 ± 0.02	135.3	−3.8**	−0.05 ± 0.02	135.3	−4.1**
Aridity	−0.8 ± 0.1	135.6	−6.5**	−0.5 ± 0.1	135.9	−5.0**	−0.35 ± 0.09	135.9	−3.7**
Microsite × Aridity									
Marginal *R* ^2^	0.21			0.16			0.10		
Conditional *R* ^2^	0.72			0.88			0.88		

* 0.01 < *p* < 0.05.

** 0.01 < *p* < 0.001.

Both taxonomic and phylogenetic diversity declined with aridity, with PD generally decreasing less steeply than TD (see slopes of diversity and aridity in Table 2). The difference between slopes increased with Hill order, being non‐significant for *q* = 0 (*t*_544_ = −0.971; *p* = 0.332), marginally significant for *q* = 1 (*t*_544_ = −1.86; *p* = 0.063), and significant for *q* = 2 (*t*_544_ = −2.24; *p* = 0.026).

Our results were robust to phylogenetic uncertainty, as the diversities obtained using the phylogeny constructed under the Jin and Qian (2019) S3 scenario were highly correlated with those obtained under the S1 (*r* = 0.9999486; *p* < 0.001) and S2 (*r* = 0.9993583; *p* < 0.001) scenarios.

## 4 Discussion

Our study provides a global synthesis of recruitment diversity patterns, showing that large‐scale climatic and local biotic factors jointly shape the taxonomic and phylogenetic diversity of recruiting species. While aridity reduces both facets of diversity at the community scale, this negative effect is locally mitigated under canopy plants, which act as custodians of taxonomic and phylogenetic diversity. Several mechanisms can be producing these canopy plant effects on the diversity of recruiting species, including the creation of a wider range of regeneration niches (Moro et al. 1997; Schöb et al. 2012), the reduction of herbivory pressure (Callaway et al. 2000), the facilitation of resource access (McIntire and Fajardo 2014), and the mitigation of competitive exclusion among recruits through indirect effects (Brooker et al. 2008).

The decline in recruitment diversity with increasing aridity is not surprising, as water availability is a key constraint on plant establishment (Kreft and Jetz 2007). Yet, the mechanisms shaping this pattern vary across diversity facets. For taxonomic diversity, *q* = 0, which gives equal weight to all species regardless of their abundance (species richness), and *q* = 2, which gives greater weight to dominant species, canopy plants consistently enhanced diversity along the aridity gradient, highlighting their protective role in maintaining both total and dominant species diversity. In contrast, for *q* = 1, which weights species in proportion to their relative abundances—giving more influence to common species while still accounting for rarer ones—a significant interaction between microsite and aridity emerged: open ground microsites showed higher diversity under low aridity, but this pattern reversed under dry conditions. This shift suggests that, as environmental stress intensifies, canopy plants promote a more even distribution of species by increasing the abundance of rare, stress‐sensitive species that benefit more from facilitative interactions under the canopy (Sánchez‐Martín et al. 2023). Such a role is consistent with the hypothesis that facilitation particularly benefits non‐dominant lineages (Soliveres et al. 2015).

Phylogenetic diversity also declined with increasing aridity, but canopy microsites consistently supported a higher number of evolutionary lineages across all Hill orders. This suggests that canopy plants act as refuges that safeguard a broader evolutionary representation within the pool of recruiting species. This greater phylogenetic diversity among recruits may result from the higher niche heterogeneity provided by canopy plants and/or from competitive exclusion processes operating among recruits (Valiente‐Banuet and Verdú 2013). The increased niche heterogeneity under canopy plants allows species with different recruitment requirements to coexist. Because these requirements are phylogenetically conserved, species occupying different niches tend to be distantly related, resulting in a neighbourhood of recruits that is more phylogenetically diverse (Navarro‐Cano et al. 2016; Paterno et al. 2016). At the same time, competitive exclusion tends to act more strongly among closely related species, so under canopy plants, the preferential survival of distantly related species further enhances the phylogenetic diversity of the recruited assemblage (Castillo et al. 2010).

Previous studies assessing the role of canopy plants have typically used different metrics for taxonomic and phylogenetic diversity, preventing direct comparisons of their relative magnitudes (Navarro‐Cano et al. 2016; Bashirzadeh et al. 2022, but see Vega‐Álvarez et al. 2019). By analysing taxonomic (TD) and phylogenetic diversity (PD) jointly using effective Hill numbers, it becomes possible to directly compare the contributions of species richness and evolutionary history to community assembly (Chao et al. 2014). We found a positive correlation between taxonomic and phylogenetic diversity that declined with Hill orders, indicating that recruiting microsites with more species also tend to host a greater number of evolutionary lineages. However, the decline in this correlation with increasing Hill order reveals that the most abundant recruits are often phylogenetically clustered—that is, dominant species belong to a limited set of related lineages. This pattern implies that while canopy and open ground microsites both harbour diverse assemblages, ecological filters such as drought tolerance, light requirements, or dispersal traits may favour recruitment of closely related taxa within a few successful clades. These results are consistent with the hierarchical filter model, in which abiotic environmental conditions act first, followed by biotic interactions (Keddy 1992; Luzuriaga et al. 2012).

Building on these patterns, we found that both taxonomic diversity (TD) and phylogenetic diversity (PD) declined with increasing aridity, although PD decreased more slowly than TD. This suggests that even as species richness is lost under drier conditions, the remaining species represent a broad range of evolutionary lineages, providing phylogenetic redundancy. Such phylogenetic, and therefore functional redundancy, is important for maintaining ecosystem functioning under ongoing species loss (Ricotta et al. 2016).

In conclusion, we show that the diversity of the pool of recruiting species depends not only on broad‐scale climatic gradients, but also on fine‐scale habitat heterogeneity. In particular, canopy plants become crucial, as they enhance not only the number of recruiting species, but also the phylogenetic history contained in the community. Current rates of climate change are outpacing the ability of many plant species to face these changes, threatening their long‐term survival. In this context, canopy plants constitute key structural elements, as they provide refuge for recruiting species experiencing high climatic disequilibrium under present macroclimatic conditions (Perez‐Navarro et al. 2024). However, climate change also pushes canopy plants outside their climatic niche optima, in turn affecting their sheltering effect on recruiting species (Margalef‐Marrase et al. 2025). Therefore, the long‐term maintenance of plant diversity under future climate change will probably depend either on the ability of canopy species to track shifting climatic conditions, or on the capacity of recruiting species to associate with more stress‐tolerant canopy plants.

## Author Contributions

M. Verdú and G. Gleiser conceived the idea; J.M. Alcántara, J.L. Garrido, A. Montesinos‐Navarro, and M. Verdú co‐ordinated the collection of data; G. Gleiser, M. Verdú and G.B. Paterno ran the statistical analyses and wrote the first draft of the manuscript; all authors contributed to subsequent revisions.

## Funding

This work was supported by the Spanish Ministry of Science, Innovation and Universities (RTI2018‐099672‐J‐I00, PID2020‐113157GB‐I00, PID2023‐146535NB‐I00, LIFEWATCH‐2019‐09‐CSIC‐4), Generalitat Valenciana (CIPROM/2021/63), the Swiss National Science Foundation (Grant 310030_197201) and by the Argentinian Fund for Research (FONCyT, PICT‐2021‐GRF‐TI‐00453).

## Supporting information


**Data S1:** Data and scripts.

**Table S2:** Pearson's pairwise correlations between taxonomic and phylogenetic Hill diversities for q0 (i.e., species abundances are not considered), q1 (i.e., greater contribution of common species to diversity estimates) and q2 (i.e., greater contribution of dominant species to diversity estimates).
**Table S3:** Comparison of the goodness of fit between linear and quadratic mixed models used to evaluate the effects of recruiting microsite and aridity on the taxonomic and phylogenetic diversity of recruiting species. Linear models included the main effects of microsite and aridity, as well as their interaction. Quadratic models additionally included a quadratic term for aridity and its interaction with microsite. Model fit was compared using likelihood ratio tests implemented with the *anova* function in R.
**Table S4:** Linear mixed models testing the effects of aridity (considered here as 1‐aridity index) and microsite (Canopy vs. open ground) and the number of canopy plants on the diversity of the plant recruits. Both the taxonomic (a) and phylogenetic (b) Hill diversities were analysed. Plant communities were incorporated in the models as a random effect. The interaction term was removed from models where it was not statistically significant.
**Figure S1:** Taxonomic (TD) and phylogenetic (PD) diversity of recruiting species across microsites at the global scale. (A). Phylogenetic trees of recruiting species occurring exclusively under canopy plants (left, green) and in open ground (right, orange). Coloured branches indicate species recruiting only in that microsite, whereas black branches represent species absent or shared between microsites. TD and PD values are shown within each tree, along with the standardized effect size of PD (SES.PD) and *p* values indicating whether observed PD is higher or lower than expected given species richness. While PD under canopy plants does not differ from random expectations, PD in open ground is marginally lower than expected. (B) Sample‐size‐based rarefaction and extrapolation curves comparing taxonomic diversity (left) and phylogenetic diversity (right) for species recruiting exclusively under canopies (green) or in open ground (orange) at *q* = 0. Solid lines represent observed and rarefied diversity, dashed lines indicate extrapolated values, and shaded areas show 95% confidence intervals (*N* = 30 randomizations).

**Table S1:** Summary of the study sites, including country, geographic coordinates, biome type, vegetation characteristics, plant community, sampling method (RN = Recruitment Network; pCO = paired Canopy–Open; GP = Georeferenced Plot), sampled area (m^2^), and the number of plant species recorded.

## Data Availability

Data on canopy‐recruit interactions used in this paper are already publicly available in Verdú et al. (2023) and also in Zenodo at https://doi.org/10.5281/zenodo.6567608. All the data and scripts to run the analyses are available as Supporting Information and also in the Zenodo repository at https://zenodo.org/records/21220756.

## References

[ele70447-bib-0001] Alcántara, J. M. , M. Pulgar , K. Trøjelsgaard , J. L. Garrido , and P. J. Rey . 2018. “Stochastic and Deterministic Effects on Interactions Between Canopy and Recruiting Species in Forest Communities.” Functional Ecology 32: 2264–2274.

[ele70447-bib-0002] Alcántara, J. M. , M. Verdú , J. L. Garrido , et al. 2025. “Key Concepts and a World‐Wide Look at Plant Recruitment Networks.” Biological Reviews 100: 1127–1151. 39727257 10.1111/brv.13177 PMC12120400

[ele70447-bib-0003] Allen, B. , M. Kon , and Y. Bar‐Yam . 2009. “A New Phylogenetic Diversity Measure Generalizing the Shannon Index and Its Application to Phyllostomid Bats.” American Naturalist 174: 236–243. 10.1086/600101 19548837

[ele70447-bib-0004] Bashirzadeh, M. , S. Soliveres , M. Farzam , and H. Ejtehadi . 2022. “Plant–Plant Interactions Determine Taxonomic, Functional and Phylogenetic Diversity in Severe Ecosystems.” Global Ecology and Biogeography 31: 649–662.

[ele70447-bib-0005] Bates, D. , M. Mächler , B. M. Bolker , and S. C. Walker . 2015. “Fitting Linear Mixed‐Effects Models Using lme4.” Journal of Statistical Software 67: 1–48.

[ele70447-bib-0006] Berdugo, M. , M. Delgado‐Baquerizo , S. Soliveres , and F. T. Maestre . 2020. “Global Ecosystem Thresholds Driven by Aridity.” Science 367: 787–790. 32054762 10.1126/science.aay5958

[ele70447-bib-0008] Brooker, R. W. , F. T. Maestre , R. M. Callaway , et al. 2008. “Facilitation in Plant Communities: The Past, the Present, and the Future.” Journal of Ecology 96: 18–34.

[ele70447-bib-0009] Callaway, R. M. 1998. “Are Positive Interactions Species‐Specific?” Oikos 82: 202–207.

[ele70447-bib-0010] Callaway, R. M. , Z. Kikvidze , and D. Kikodze . 2000. “Facilitation by Unpalatable Weeds May Conserve Plant Diversity in Overgrazed Meadows in the Caucasus Mountains.” Oikos 89: 275–282.

[ele70447-bib-0012] Castillo, J. P. , M. Verdú , and A. Valiente‐Banuet . 2010. “Neighborhood Phylodiversity Affects Plant Performance.” Ecology 91: 3656–3663. 21302836 10.1890/10-0720.1

[ele70447-bib-0013] Chao, A. , C. H. Chiu , and L. Jost . 2010. “Phylogenetic Diversity Measures Based on Hill Numbers.” Philosophical Transactions of the Royal Society B: Biological Sciences 365: 3599–3609. 10.1098/rstb.2010.0272 PMC2982003 20980309

[ele70447-bib-0014] Chao, A. , C.‐H. Chiu , and L. Jost . 2014. “Unifying Species Diversity, Phylogenetic Diversity, Functional Diversity, and Related Similarity and Differentiation Measures Through Hill Numbers.” Annual Review of Ecology, Evolution, and Systematics 45: 297–324.

[ele70447-bib-0015] Chao, A. , P. A. Henderson , C.‐H. Chiu , et al. 2021. “Measuring Temporal Change in Alpha Diversity: A Framework Integrating Taxonomic, Phylogenetic and Functional Diversity and the iNEXT.3D Standardization.” Methods in Ecology and Evolution 12: 1926–1940.

[ele70447-bib-0016] Chao, A. , and L. Jost . 2012. “Coverage‐Based Rarefaction and Extrapolation: Standardizing Samples by Completeness Rather Than Size.” Ecology 93: 2533–2547. 23431585 10.1890/11-1952.1

[ele70447-bib-0017] Devictor, V. , D. Mouillot , C. Meynard , F. Jiguet , W. Thuiller , and N. Mouquet . 2010. “Spatial Mismatch and Congruence Between Taxonomic, Phylogenetic and Functional Diversity: The Need for Integrative Conservation Strategies in a Changing World.” Ecology Letters 13: 1030–1040. 20545736 10.1111/j.1461-0248.2010.01493.x

[ele70447-bib-0018] Fagundes, M. V. , R. S. Oliveira , C. R. Fonseca , and G. Ganade . 2022. “Nurse‐Target Functional Match Explains Plant Facilitation Strength.” Flora 292: 152061.

[ele70447-bib-0019] Faith, D. P. 1992. “Conservation Evaluation and Phylogenetic Diversity.” Biological Conservation 61: 1–10.

[ele70447-bib-0020] Franklin, J. , J. M. Serra‐Diaz , A. D. Syphard , and H. M. Regan . 2016. “Global Change and Terrestrial Plant Community Dynamics.” Proceedings of the National Academy of Sciences of the United States of America 113: 3725–3734. 26929338 10.1073/pnas.1519911113 PMC4833242

[ele70447-bib-0021] Gleiser, G. , J. M. Alcántara , J. Bascompte , et al. 2025. “The Phylogenetic Architecture of Recruitment Networks.” Global Ecology and Biogeography 34: 1–11.

[ele70447-bib-0022] Gómez‐Aparicio, L. 2008. “Spatial Patterns of Recruitment in Mediterranean Plant Species: Linking the Fate of Seeds, Seedlings and Saplings in Heterogeneous Landscapes at Different Scales.” Journal of Ecology 96: 1128–1140.

[ele70447-bib-0023] Grubb, P. J. 1977. “The Maintenance of Species‐Richness in Plant Communities: The Importance of the Regeneration Niche.” Biological Reviews 52: 107–145.

[ele70447-bib-0024] Hähn, G. J. A. , G. Damasceno , E. Alvarez‐Davila , et al. 2024. “Global Decoupling of Functional and Phylogenetic Diversity in Plant Communities.” Nature Ecology & Evolution 9: 237–248. 39627407 10.1038/s41559-024-02589-0

[ele70447-bib-0025] Harper, J. L. 1977. Population Biology of Plants. Academic Press.

[ele70447-bib-0026] Harrison, S. , M. J. Spasojevic , and D. Li . 2020. “Climate and Plant Community Diversity in Space and Time.” Proceedings of the National Academy of Sciences of the United States of America 117: 4464–4470. 32071212 10.1073/pnas.1921724117 PMC7060689

[ele70447-bib-0027] Hautier, Y. , F. Isbell , E. T. Borer , et al. 2018. “Local Loss and Spatial Homogenization of Plant Diversity Reduce Ecosystem Multifunctionality.” Nature Ecology & Evolution 2: 50–56. 29203922 10.1038/s41559-017-0395-0

[ele70447-bib-0028] Hawkins, B. A. , R. Field , H. V. Cornell , D. J. Currie , J.‐F. Guégan , and D. M. Kaufman . 2003. “Energy, Water, and Broad‐Scale Geographic Patterns of Species Richness.” Ecology 84: 3105–3117.

[ele70447-bib-0075] Hijmans, R. J. 2023. raster: Geographic Data Analysis and Modeling. R package version 3.6‐20. https://CRAN.R‐project.org/package=raster.

[ele70447-bib-0029] Hill, M. O. 1973. “Diversity and Evenness: A Unifying Notation and Its Consequences.” Ecology 54: 427–432.

[ele70447-bib-0030] Jin, Y. , and H. Qian . 2019. “V.PhyloMaker: An R Package That Can Generate Very Large Phylogenies for Vascular Plants.” Ecography 42: 1353–1359. 10.1016/j.pld.2022.05.005 PMC9363651 35967255

[ele70447-bib-0031] Jin, Y. , and H. Qian . 2022. “V.PhyloMaker2: An Updated and Enlarged R Package That Can Generate Very Large Phylogenies for Vascular Plants.” Plant Diversity 44: 335–339. 35967255 10.1016/j.pld.2022.05.005 PMC9363651

[ele70447-bib-0032] Karger, D. N. , O. Conrad , J. Böhner , et al. 2021. “Climatologies at High Resolution for the Earth's Land Surface Areas.” Scientific Data 4: 170122. 10.1038/sdata.2017.122 PMC5584396 28872642

[ele70447-bib-0033] Keddy, P. A. 1992. “Assembly and Response Rules: Two Goals for Predictive Community Ecology.” Journal of Vegetation Science 3: 157–164.

[ele70447-bib-0034] Kembel, S. W. , P. D. Cowan , M. R. Helmus , et al. 2010. “Picante: R Tools for Integrating Phylogenies and Ecology.” Bioinformatics 26: 1463–1464. 20395285 10.1093/bioinformatics/btq166

[ele70447-bib-0035] Kreft, H. , and W. Jetz . 2007. “Global Patterns and Determinants of Vascular Plant Diversity.” Proceedings of the National Academy of Sciences of the United States of America 104: 5925–5930. 17379667 10.1073/pnas.0608361104 PMC1851593

[ele70447-bib-0036] Kuznetsova, A. , P. B. Brockhoff , and R. H. B. Christensen . 2017. “lmerTest Package: Tests in Linear Mixed Effects Models.” Journal of Statistical Software 82: 1–26.

[ele70447-bib-0038] Luzuriaga, A. L. , A. M. Sánchez , F. T. Maestre , and A. Escudero . 2012. “Assemblage of a Semi‐Arid Annual Plant Community: Abiotic and Biotic Filters Act Hierarchically.” PLoS One 7: e41270. 22848455 10.1371/journal.pone.0041270 PMC3407229

[ele70447-bib-0039] Maestre, F. T. , R. M. Callaway , F. Valladares , and C. J. Lortie . 2009. “Refining the Stress‐Gradient Hypothesis for Competition and Facilitation in Plant Communities.” Journal of Ecology 97: 199–205.

[ele70447-bib-0040] Margalef‐Marrase, J. , F. Lloret , A. Montesinos‐Navarro , J. M. Alcántara , J. L. Garrido , and M. Verdú . 2025. “Climatic Disequilibrium Modulates Canopy Service Across Abiotic Stress Gradients.” Journal of Ecology 113: 2160–2172.

[ele70447-bib-0041] May, F. E. , and J. E. Ash . 1990. “An Assessment of the Allelopathic Potential of Eucalyptus.” Australian Journal of Botany 38: 245–254.

[ele70447-bib-0042] McIntire, E. J. B. , and A. Fajardo . 2014. “Facilitation as a Ubiquitous Driver of Biodiversity.” New Phytologist 201: 403–416. 24102266 10.1111/nph.12478

[ele70447-bib-0043] Miriti, M. N. 2006. “Ontogenetic Shift From Facilitation to Competition in a Desert Shrub.” Journal of Ecology 94: 973–979.

[ele70447-bib-0044] Mirzabaev, A. , J. Wu , J. Evans , et al. 2019. “Desertification.” In Climate Change and Land: An IPCC Special Report on Climate Change, Desertification, Land Degradation, Sustainable Land Management, Food Security, and Greenhouse Gas Fluxes in Terrestrial Ecosystems, edited by P. R. Shukla , J. Skea , E. C. Buendia , et al., 249–343. Cambridge University Press.

[ele70447-bib-0045] Moro, M. J. , F. I. Pugnaire , P. Haase , and J. Puigdefàbregas . 1997. “Mechanisms of Interaction Between a Leguminous Shrub and Its Understorey in a Semi‐Arid Environment.” Ecography 20: 175–184.

[ele70447-bib-0046] Navarro‐Cano, J. A. , P. P. Ferrer‐Gallego , E. Laguna , et al. 2016. “Restoring Phylogenetic Diversity Through Facilitation.” Restoration Ecology 24: 449–455.

[ele70447-bib-0047] O'Brien, M. J. , L. F. T. de Menezes , K. A. Bråthen , G. Losapio , and F. I. Pugnaire . 2019. “Facilitation Mediates Species Presence Beyond Their Environmental Optimum.” Perspectives in Plant Ecology, Evolution and Systematics 38: 24–30.

[ele70447-bib-0048] Paterno, G. B. , J. A. J. A. Siqueira Filho , and G. Ganade . 2016. “Species‐Specific Facilitation, Ontogenetic Shifts and Consequences for Plant Community Succession.” Journal of Vegetation Science 27: 606–615.

[ele70447-bib-0049] Pausas, J. G. , and J. E. Keeley . 2021. “Wildfires and Global Change.” Frontiers in Ecology and the Environment 19: 387–395.

[ele70447-bib-0050] Perez‐Navarro, M. A. , F. Lloret , R. Molina‐Venegas , J. M. Alcántara , and M. Verdú . 2024. “Plant Canopies Promote Climatic Disequilibrium in Mediterranean Recruit Communities.” Ecology Letters 27: e14391. 38400769 10.1111/ele.14391

[ele70447-bib-0076] R Core Team 2024. R: A Language and Environment for Statistical Computing. R Foundation for Statistical Computing. https://www.R‐project.org/.

[ele70447-bib-0052] Rao, R. C. 1982. “Diversity and Dissimilarity Coefficients: A Unified Approach.” Theoretical Population Biology 21: 24–43.

[ele70447-bib-0054] Ricotta, C. , F. de Bello , M. Moretti , M. Caccianiga , B. E. Cerabolini , and S. Pavoine . 2016. “Measuring the Functional Redundancy of Biological Communities: A Quantitative Guide.” Methods in Ecology and Evolution 7: 1386–1395.

[ele70447-bib-0055] Sánchez‐Martín, R. , A. Montesinos‐Navarro , H. Ochoterena , et al. 2024. “Homogeneous Microenvironmental Conditions Under Nurses Promote Facilitation.” Functional Ecology 38: 350–362.

[ele70447-bib-0056] Sánchez‐Martín, R. , M. Verdú , and A. Montesinos‐Navarro . 2023. “Phylogenetic and Functional Constraints of Plant Facilitation Rewiring.” Ecology 104: e3961. 36545892 10.1002/ecy.3961 PMC10078402

[ele70447-bib-0057] Schöb, C. , B. J. Butterfield , and F. I. Pugnaire . 2012. “Foundation Species Influence Trait‐Based Community Assembly.” New Phytologist 196: 824–834. 22978646 10.1111/j.1469-8137.2012.04306.x

[ele70447-bib-0058] Shackelford, N. , G. B. Paterno , D. E. Winkler , et al. 2021. “Drivers of Seedling Establishment Success in Dryland Restoration Efforts.” Nature Ecology & Evolution 5: 1283–1290. 34294898 10.1038/s41559-021-01510-3

[ele70447-bib-0059] Smith, S. A. , and J. W. Brown . 2018. “Constructing a Broadly Inclusive Seed Plant Phylogeny.” American Journal of Botany 105: 302–314. 29746720 10.1002/ajb2.1019

[ele70447-bib-0060] Soliveres, S. , D. J. Eldridge , F. T. Maestre , et al. 2011. “Microhabitat Amelioration and Reduced Competition Among Understorey Plants as Drivers of Facilitation Across Environmental Gradients: Towards a Unifying Framework.” Perspectives in Plant Ecology, Evolution and Systematics 13: 247–258. 25914601 10.1016/j.ppees.2011.06.001 PMC4407968

[ele70447-bib-0061] Soliveres, S. , C. Smit , and F. T. Maestre . 2014. “Moving Forward on Facilitation Research: Response to Changing Environments and Species Interactions.” Journal of Ecology 102: 1–5.

[ele70447-bib-0062] Soliveres, S. , C. Smit , and F. T. Maestre . 2015. “Moving Forward on Facilitation Research: Response to Changing Environments and Effects on the Diversity, Functioning and Evolution of Plant Communities.” Biological Reviews of the Cambridge Philosophical Society 90: 297–313. 24774563 10.1111/brv.12110 PMC4407973

[ele70447-bib-0063] Swenson, N. G. 2011. “The Role of Evolutionary Processes in Producing Biodiversity Patterns, and the Interrelationships Between Taxonomic, Functional and Phylogenetic Biodiversity.” American Journal of Botany 98: 472–480. 21613140 10.3732/ajb.1000289

[ele70447-bib-0064] Tucker, C. M. , T. J. Davies , M. W. Cadotte , and W. D. Pearse . 2018. “On the Relationship Between Phylogenetic Diversity and Trait Diversity.” Ecology 99: 1473–1479. 29782644 10.1002/ecy.2349

[ele70447-bib-0065] Valdez, J. W. , F. Hartig , S. Fennel , and P. Poschlod . 2019. “The Recruitment Niche Predicts Plant Community Assembly Across a Hydrological Gradient Along Plowed and Undisturbed Transects in a Former Agricultural Wetland.” Frontiers in Plant Science 10: 1–9. 30787938 10.3389/fpls.2019.00088 PMC6372561

[ele70447-bib-0066] Valiente‐Banuet, A. , and M. Verdú . 2007. “Facilitation Can Increase the Phylogenetic Diversity of Plant Communities.” Ecology Letters 10: 1029–1036. 17714492 10.1111/j.1461-0248.2007.01100.x

[ele70447-bib-0067] Valiente‐Banuet, A. , and M. Verdú . 2013. “Plant Facilitation and Phylogenetics.” Annual Review of Ecology, Evolution, and Systematics 44: 347–366.

[ele70447-bib-0068] Vega‐Álvarez, J. , J. A. García‐Rodríguez , and L. Cayuela . 2019. “Facilitation Beyond Species Richness.” Journal of Ecology 107: 722–734.

[ele70447-bib-0069] Verdú, M. , J. L. Garrido , J. M. Alcántara , et al. 2023. “RecruitNet: A Global Database of Plant Recruitment Networks.” Ecology 104: 1–5. 10.1002/ecy.3923 PMC10078134 36428233

[ele70447-bib-0070] Wagg, C. , M. J. O'Brien , A. Vogel , et al. 2017. “Plant Diversity Maintains Long‐Term Ecosystem Productivity Under Frequent Drought by Increasing Short‐Term Variation.” Ecology 98: 2952–2961. 28869781 10.1002/ecy.2003

[ele70447-bib-0071] Walck, J. L. , S. N. Hidayati , K. W. Dixon , K. Thompson , and P. Poschlod . 2011. “Climate Change and Plant Regeneration From Seed.” Global Change Biology 17: 2145–2161.

[ele70447-bib-0072] Weedon, J. T. , and J. M. Facelli . 2008. “Desert Shrubs Have Negative or Neutral Effects on Annuals at Two Levels of Water Availability in Arid Lands of South Australia.” Journal of Ecology 96: 1230–1237.

[ele70447-bib-0073] WFO . 2023. “World Flora Online.” https://www.worldfloraonline.org.

[ele70447-bib-0074] Yang, X. , L. Gómez‐Aparicio , C. J. Lortie , et al. 2022. “Net Plant Interactions Are Highly Variable and Weakly Dependent on Climate at the Global Scale.” Ecology Letters 25: 1580–1593. 35460586 10.1111/ele.14010

