# Supplementary material for: Canopy Plants Safeguard the Taxonomic and Phylogenetic Diversity of Recruiting Plants Worldwide
Source: Ecol Lett. 2026 Aug 25;29(8):e70447. doi: 10.1111/ele.70447 (PMC13506208; doi:10.1111/ele.70447)
Supplement: Supplementary file 1 [file ELE-29-0-s003.docx]

Table S2. Pearson’s pairwise correlations between taxonomic and phylogenetic Hill diversities for q0 (i.e., species abundances are not considered), q1 (i.e., greater contribution of common species to diversity estimates) and q2 (i.e., greater contribution of dominant species to diversity estimates).

|  | Open ground | | Under canopies | |
| --- | --- | --- | --- | --- |
| Hill order | *r* | *p* | *r* | *p* |
| q0 | 0.874 | <0.001 | 0.859 | <0.001 |
| q1 | 0.629 | <0.001 | 0.528 | <0.001 |
| q2 | 0.474 | <0.001 | 0.352 | <0.001 |

**Table S3.** Comparison of the goodness of fit between linear and quadratic mixed models used to evaluate the effects of recruiting microsite and aridity on the taxonomic and phylogenetic diversity of recruiting species. Linear models included the main effects of microsite and aridity, as well as their interaction. Quadratic models additionally included a quadratic term for aridity and its interaction with microsite. Model fit was compared using likelihood ratio tests implemented with the *anova* function in R.

|  |  | Linear model | | Quadratic model | |  |  |
| --- | --- | --- | --- | --- | --- | --- | --- |
| Diversity | Hill order | AIC | Log-likelihood | AIC | Log-likelihood | χ^2^ | p |
| Taxonomic | q0 | 685.3 | -336.6 | 690.2 | -337.1 | 0.5 | 0.78 |
|  | q1 | 556.1 | -272.1 | 560.0 | -272.0 | 2.5 | 0.29 |
|  | q2 | 634.6 | -311.3 | 636.5 | -310.3 | 3.9 | 0.14 |
| Phylogenetic | q0 | 463.1 | -225.5 | 469.9 | -226.9 | 0.1 | 0.96 |
|  | q1 | 263.9 | -126.0 | 270.2 | -127.1 | 2.2 | 0.34 |
|  | q2 | 200.7 | -94.3 | 204.9 | -94.5 | 4.7 | 0.09 |

Table S4. Linear mixed models testing the effects of aridity (considered here as 1-aridity index) and microsite (Canopy vs open ground) and the number of canopy plants on the diversity of the plant recruits. Both the taxonomic (a) and phylogenetic (b) Hill diversities were analysed. Plant communities were incorporated in the models as a random effect. The interaction term was removed from models where it was not statistically significant.

|  | Taxonomic Diversity | | | | | | | | | | | |
| --- | --- | --- | --- | --- | --- | --- | --- | --- | --- | --- | --- | --- |
|  | q=0 | | |  | q=1 | | |  | | q=2 | | |
|  | Est ±SE | df | t |  | Est ±SE | df | t |  | | Est ±SE | df | t |
| Intercept | 3.4±0.1 | 175.4 | 27.1^**^ |  | 2.3±0.1 | 177 | 20.1^**^ |  | | 1.9±0.1 | 160.4 | 16.3^**^ |
| Microsite (Open ground) | -0.3±0.1 | 141 | -4.1^**^ |  | -0.1±0.08 | 140 | -1.6 |  | | -0.1±0.1 | 141 | -2.4^*^ |
| Aridity | -0.5±0.2 | 140 | -3.2^*^ |  | -0.3±0.2 | 183 | -1.6^*^ |  | | -0.3±0.2 | 139 | -1.7^ms^ |
| Canopy sp | 0.006±0.001 | 140 | 5.1^**^ |  | 0.01±0.001 | 139 | 5.1^**^ |  | | 0.01±0.001 | 139 | 4.3^**^ |
| Microsite  x Aridity |  |  |  |  | -0.3±0.1 | 140 | -2.1^*^ |  | |  |  |  |
| Marginal R^2^ | 0.30  0.60 | | |  | 0.30  0.73 | | |  | | 0.19  0.59 | | |
| Conditional R^2^ |  |  |  |  |  |  |  |  | |  |  |  |
| ms 0.05<p<0.1; *0.01<p<0.05; ** 0.01<p<0.001 | | | | | | | | | | | | |
|  | | |  |  |  |  |  |  | |  |  |  |
| (b) |  |  |  |  |  |  |  |  | |  |  |  |
|  | Phylogenetic Diversity | | | | | | | | | | | |
|  | q=0 | | |  | q=1 | | |  | | q=2 | | |
|  | Est ±SE | df | t |  | Est ±SE | df | t |  | Est ±SE | | df | t |
| Intercept | 2.5±0.1 | 149 | 25.14^**^ |  | 1.6±0.09 | 139.6 | 17.5^**^ |  | 1.2±0.1 | | 139.1 | 15.4^**^ |
| Microsite (Open ground) | -0.1±0.04 | 135.6 | -3.0^**^ |  | -0.09±0.02 | 135.3 | -4.1^**^ |  | -0.07±0.02 | | 135.3 | -3.5^**^ |
| Aridity | -0.5±0.1 | 134.6 | -3.6^**^ |  | -0.5±0.1 | 135.0 | -4.2^**^ |  | -0.46±0.11 | | 134.9 | -4.1^**^ |
| Canopy sp | 3e03±9e04 | 134.2 | 3.7^**^ |  | 4e-05±8e-04 | 134.8 | 0.04 |  | -1e03±7e04 | | 134.8 | -1.6 |
| Microsite  x Aridity |  |  |  |  |  |  |  |  |  | |  |  |
| Marginal R^2^ | 0.27  0.72 | | |  | 0.16  0.88 | | |  | | 0.11  0.89 | | |
| Conditional R^2^ |  |  |  |  |  |  |  |  | |  |  |  |
| *0.01<p<0.05; ** 0.01<p<0.001 | | | | | | | | | | | | |

**Figure S1.** Taxonomic (TD) and phylogenetic (PD) diversity of recruiting species across microsites at the global scale. **(A)**. Phylogenetic trees of recruiting species occurring exclusively under canopy plants (left, green) and in open ground (right, orange). Coloured branches indicate species recruiting only in that microsite, whereas black branches represent species absent or shared between microsites. TD and PD values are shown within each tree, along with the standardized effect size of PD (SES.PD) and p-values indicating whether observed PD is higher or lower than expected given species richness. While PD under canopy plants does not differ from random expectations, PD in open ground is marginally lower than expected. **(B)** Sample-size–based rarefaction and extrapolation curves comparing taxonomic diversity (left) and phylogenetic diversity (right) for species recruiting exclusively under canopies (green) or in open ground (orange) at q = 0. Solid lines represent observed and rarefied diversity, dashed lines indicate extrapolated values, and shaded areas show 95% confidence intervals (N = 30 randomizations).
