# Supplementary material for: Canopy Plants Safeguard the Taxonomic and Phylogenetic Diversity of Recruiting Plants Worldwide
Source: Ecol Lett. 2026 Aug 25;29(8):e70447. doi: 10.1111/ele.70447 (PMC13506208; doi:10.1111/ele.70447)
Supplement: Supplementary file 2 [file ELE-29-0-s001.zip › Data and scripts/README.docx]

**README RecruitNet_Hill.xlsx**

**Dataset description**

**RecruitNet_Hill.xlsx** contains records of plant recruitment interactions observed across study sites worldwide. Each row represents the abundance of one recruiting species associated with one canopy species (or open ground) within a sampling plot, together with site characteristics and sampling metadata.

| **Variable** | **Description** |
| --- | --- |
| **Study_site** | Unique identifier of each recruitment network (study site). |
| **Location** | Geographic location of the study site. |
| **Site_responsible** | Name of the principal investigator responsible for data collection. |
| **Country** | Country where the study site was sampled. |
| **Biome** | Biome classified according to the Major Habitat Types of Olson et al. (2001. *BioScience*, 5:1 933–938). |
| **Vegetation_type** | Vegetation type where the recruitment network was sampled. |
| **Community** | Name of the plant community. |
| **Successional_stage** | Successional stage of the community (Early, Mid, or Late). |
| **Disturbance** | Main disturbance(s) affecting the study site (e.g., browsing, fire, agriculture, grazing). Multiple disturbances may be listed. |
| **Disturbance_ID** | Disturbance category: 1 = Human disturbances; 2 = Climatic or stochastic events; 3 = No disturbance. |
| **Latitude** | Latitude of the sampling plot (decimal degrees, WGS84). |
| **Longitude** | Longitude of the sampling plot (decimal degrees, WGS84). |
| **PlotdimX** | Plot length (m). |
| **PlotdimY** | Plot width (m). |
| **Sampling_date** | Year when sampling was conducted. |
| **Plot** | Unique identifier of the sampling plot within each study site. |
| **Canopy** | Scientific name of the canopy (potential nurse) species, or "Open" when the recruit occurred in an open microsite. |
| **Recruit** | Scientific name of the recruiting species. |
| **Standardized_Canopy** | Taxonomically standardized canopy species name according to the World Flora Online and Missouri Botanical Garden Tropicos databases. |
| **Standardized_Recruit** | Taxonomically standardized recruit species name according to the World Flora Online and Missouri Botanical Garden Tropicos databases. |
| **Family_Canopy** | Taxonomic family of the canopy species. |
| **Family_Recruit** | Taxonomic family of the recruiting species. |
| **Frequency** | Number of individuals of the recruit species recorded beneath individuals of the corresponding canopy species within the sampling plot. |
| **Sampling_method** | Sampling protocol used to construct the recruitment network: RN = Recruitment Network; pCO = Paired Canopy–Open sampling; GP = Georeferenced Plots. |
| **LifeHabit_Canopy** | Growth form of the canopy species (Woody, Herb, or Variable). |
| **LifeHabit_Recruit** | Growth form of the recruiting species (Woody, Herb, or Variable). |

**README – traits.txt**

**Dataset description**

**traits.txt** contains site- and network-level descriptors for each plant recruitment network included in the database. Each row represents one recruitment network (study site) and includes information on network composition, geographic location, climate, disturbance regime, sampling methodology, and summary metrics describing plant recruitment interactions.

| **Variable** | **Description** |
| --- | --- |
| **Study_site** | Unique identifier of the recruitment network (study site). |
| **nb_A** | Number of canopy (potential nurse) species recorded in the network. |
| **nb_B** | Number of recruiting species recorded in the network. |
| **aridity** | Aridity index of the study site obtained from Karger et al. 2001. Scientific Data 4: 170122.  Resolution for the Earth's Land Surface Areas.” Scientific Data 4: 170122. |
| **bio_1** | Annual Mean Temperature (BIO1, CHELSA). Values are stored as temperature × 10. |
| **bio_3** | Isothermality (BIO3, CHELSA). |
| **bio_4** | Temperature Seasonality (BIO4, CHELSA). |
| **bio_5** | Maximum Temperature of the Warmest Month (BIO5, CHELSA). Values are stored as temperature × 10. |
| **bio_6** | Minimum Temperature of the Coldest Month (BIO6, CHELSA). Values are stored as temperature × 10. |
| **bio_7** | Annual Temperature Range (BIO7, CHELSA). |
| **bio_12** | Annual Precipitation (BIO12, CHELSA; mm). |
| **bio_14** | Precipitation of the Driest Month (BIO14, CHELSA; mm). |
| **bio_15** | Precipitation Seasonality (BIO15, CHELSA; coefficient of variation). |
| **bio_17** | Precipitation of the Driest Quarter (BIO17, CHELSA; mm). |
| **bio_18** | Precipitation of the Warmest Quarter (BIO18, CHELSA; mm). |
| **gsp** | Growing Season Precipitation (mm). |
| **npp** | Net Primary Productivity. |
| **hurs_min** | Minimum relative humidity. |
| **growing_season_length** | Length of the growing season (days). |
| **Lat_mean** | Mean latitude of the study site (decimal degrees, WGS84). |
| **long_mean** | Mean longitude of the study site (decimal degrees, WGS84). |
| **Disturbance** | Disturbance category: 1 = Human disturbances; 2 = Climatic or stochastic events; 3 = No disturbance. |
| **successional_stage** | Successional stage of the vegetation (Early, Mid, or Late). |
| **sampling_method** | Sampling protocol used to construct the recruitment network: RN = Recruitment Network; pCO = Paired Canopy–Open sampling; GP = Georeferenced Plots. |
| **stdres** | Standardized residual of the canopy–recruit association test. Residual values are provided for all statistically testable networks and quantify the direction of the association,as explained in the testability variable. |
| **testability** | Indicates whether the canopy–recruit association could be statistically evaluated given the available sample size. NA indicates that the association was not testable because of insufficient sampling. For testable networks, the association is classified as Positive, Negative, or Neutral according to the significance and direction of the standardized residual. Neutral associations may therefore have either positive or negative standardized residuals that do not reach the significance threshold. |
| **RII** | Relative Interaction Index (RII), quantifying the net effect of canopy plants on recruitment. Calculated following Armas et al. (2004. *Ecology* 85: 2682–2686). |
| **N_rec** | Number of recruiting species recorded in the network. |
| **N_rec_facil** | Number of recruiting species classified as positively associated with canopy plants. |
| **N_rec_negat** | Number of recruiting species classified as negatively associated with canopy plants. |
| **N_inter** | Total number of canopy–recruit interactions recorded in the network. |
| **N_rec_pos_inter** | Number of positive canopy–recruit interactions. |
| **N_rec_negat_inter** | Number of negative canopy–recruit interactions. |
